# Multi-Function Computation over a Directed Acyclic Network

**DOI:** 10.3390/e27121225

**Published:** 2025-12-03

**Authors:** Xiufang Sun, Ruze Zhang, Dan Li, Xuan Guang

**Affiliations:** 1 School of Mathematical Sciences and LPMC, Nankai University, Tianjin 300071, China; xfsun@mail.nankai.edu.cn; 2 Institute of Network Coding, The Chinese University of Hong Kong, Hong Kong SAR, China; rzzhang@inc.cuhk.edu.hk; 3 School of Science, Tianjin University of Technology, Tianjin 300384, China; lidaneileen@163.com

**Keywords:** network multi-function computation, vector-linear function, rate region, strong partition, network function computation

## Abstract

The problem of multi-function computation over a directed acyclic network is investigated in this paper. In such a network, a sink node is required to compute with zero error multiple vector-linear functions, where each vector-linear function has distinct inputs generated by multiple source nodes. The *computing rate tuple* of an admissible code is defined as a tuple consisting of the average number of zero-error computations for each vector-linear function when the network is used once jointly. From the information theoretic point of view, we are interested in characterizing the *rate region*, which is defined as the closed set of all achievable computing rate tuples. In particular, when the sink node is required to compute a single vector-linear function, the *network multi-function computation* problem degenerates to the *network function computation* problem. We prove an outer bound on the rate region by developing the approach of the cut-set strong partition. We also illustrate that the obtained outer bound is tight for a typical model of computing two vector-linear functions over the diamond network. Furthermore, we establish the relationship between the network multi-function computation rate region and the network function computation rate region. Also, we show that the best known outer bound on the rate region for computing an arbitrary vector-linear function over an arbitrary network is a straightforward consequence of our outer bound.

## 1. Introduction

In this paper, we consider the problem of multi-function computation over a directed acyclic network, called network multi-function computation. A directed acyclic graph is used to model the network, where some nodes are referred to as *source nodes*, and another node is referred to as the *sink node*. The sink node is required to compute with zero error multiple vector-linear functions, where each vector-linear function has distinct inputs generated by the source nodes. The computing rate tuple of an admissible code is defined as a tuple consisting of the average number of zero-error computations for each vector-linear function when the network is used once jointly. From the information theoretic point of view, we are interested in characterizing the rate region, which is defined as the closed set of all achievable computing rate tuples. This rate region measures the efficiency of computing the vector-linear functions over the network. We note that when the sink node is required to compute a single vector-linear function, the network multi-function computation problem degenerates to the network function computation problem (cf. [1,2,3,4,5,6,7,8,9,10,11,12,13,14,15,16]). The multi-function computation problems have been extensively studied in the literature, e.g., [17,18,19,20,21,22,23,24,25,26,27], as multiple tasks often need to be jointly performed on a single device or within a shared communication infrastructure.

The model of network function computation for computing a target function over a directed acyclic network has been investigated persistently in the literature [1,2,3,4,5,6,7,8,9,10,11,12,13,14,15,16]. Appuswamy et al. [1] investigated the fundamental *computing capacity*, i.e., the maximum average number of times that the target function can be computed with zero error for one use of the network, and gave a cut-set based upper bound that is valid under certain constraints on either the network topology or the target function. Huang et al. [2] enhanced Appuswamy et al.’s upper bound that can be applied for arbitrary functions and arbitrary network topologies. Furthermore, for the case of computing an arbitrary function over a multi-edge tree network and the case of computing the identity function or the algebraic sum over an arbitrary network topology, the above two upper bounds coincide and are tight (see [1,2]). Appuswamy and Franceschetti [3] investigated the solvability (rate-1 achievability) of linear (function-computing) network codes for computing a vector-linear function over a directed acyclic network. Subsequently, Guang et al. [4] proved an improved general upper bound by using a novel approach of the cut-set strong partition, which is not only a strict improvement over the previous upper bounds but also tight for all the considered network function computation problems previous to [4], whose computing capacities are known. In particular, the improved upper bound was used to enhance the results in [4] for computing a vector-linear function over a directed acyclic network. Based on the improved upper bound, Li and Xu [12] characterized the computing capacity for computing an arbitrary vector-linear function over the diamond network.

The main contributions and organization of the paper are given as follows.

- In Section 2, we formally present the model of network multi-function computation, and define the network multi-function computing codes and the rate region.
- In Section 3, we prove an outer bound on the rate region by developing the approach of the cut-set strong partition introduced by Guang et al. [4], which is applicable to arbitrary network topologies and arbitrary vector-linear functions. We also illustrate that the obtained outer bound is tight for a typical model of computing two vector-linear functions over the diamond network.
- In Section 4, we compare network multi-function computation and network function computation. We first establish the relationship between the network multi-function computation rate region and the network function computation rate region. By this relationship, we show that the best known outer bound in [4] on the network function computation rate region can induce an outer bound on the network multi-function computation rate region. However, this induced outer bound is not as tight as our outer bound. Further, we show that the best known outer bound in [4] on the rate region for computing an arbitrary vector-linear function over an arbitrary network is a straightforward consequence of our outer bound.
- Finally, we conclude in Section 5 with a summary of our results.

## 2. Preliminaries

### 2.1. Model of Network Multi-Function Computation

Let G=(V,E) be a directed acyclic graph, where V is a finite node set and E is a finite edge set. For an edge e∈E connecting a node u∈V to another node v∈V, we use tail(e) and head(e) to denote the *tail* node and the *head* node of *e*, respectively, i.e., u=tail(e) and v=head(e). Accordingly, for a node v∈V, let

$$\mathrm{In}\left(v\right)=\left\{e\in E:\mathrm{head}(e)=v\right\}\;\mathrm{and}\;\mathrm{Out}\left(v\right)=\left\{e\in E:\mathrm{tail}(e)=v\right\},$$

the set of input edges of *v* and the set of output edges of *v*, respectively. In the graph G, there is a set of source nodes $S=\{\sigma_{1},\sigma_{2},\ldots ,\sigma_{s}\}\subset V$ with |S|=s, and a single sink node ρ∈V∖S, where each source node has no input edges and the sink node ρ has no output edges, i.e., $\mathrm{In}\left(\sigma_{i}\right)=\mathrm{Out}\left(\rho \right)=\emptyset$ for i=1,2,…,s. Without loss of generality, we assume that for each v∈V∖{ρ}, there always exists a directed path from *v* to ρ in G. Let $F_{q}$ be a finite field of size *q*. We allow multiple edges between two nodes and assume that a symbol taken from the finite field $F_{q}$ can be transmitted with zero error on each edge for each use. The graph G, together with *S* and ρ, forms a network N, i.e., N=(G,S,ρ).

Consider *t* nonnegative integers $k_{1},k_{2},\ldots ,k_{t}$. We assume that each source node $\sigma_{i}\in S$ generates *t* sequences $x_{i,1},x_{i,2},\ldots ,x_{i,t}$. Specifically, for each index *j* with 1≤j≤t, the sequence $x_{i,j}$ is defined as an $F_{q}$-valued column $k_{j}$-vector, i.e., $x_{i,j}\in F_{q}^{k_{j}}$, called the *j-th source sub-vector* generated by $\sigma_{i}$. Let $k≜\sum_{j=1}^{t}k_{j}.$ Then, all the *t* sequences generated by the source node $\sigma_{i}\in S$ can be further written a column k-vector:

$$\begin{array}{c} x_{i}≜\left[\begin{array}{c} x_{i,1}\\ x_{i,2}\\ \vdots \\ x_{i,t} \end{array}\right]\in F_{q}^{\sum_{j=1}^{t}k_{j}}=F_{q}^{k}, \end{array}$$

called the *source vector* generated by $\sigma_{i}$.

For a subset of source nodes I⊆S, we let

$$\begin{array}{c} x_{I,j}≜\left(x_{i,j}:\;\sigma_{i}\in I\right)\in F_{q}^{k_{j}\times \left|I\right|},\;\forall 1\leq j\leq t, \end{array}$$

where $x_{I,j}$ is called *the j-th source submatrix* generated by the source nodes in *I*. In the rest of the paper, we use $F_{q}^{k_{j}\times I}$ (instead of $F_{q}^{k_{j}\times \left|I\right|}$ for notational simplicity) to denote the set of all possible $k_{j}\times \left|I\right|$ matrices taken by $x_{I,j}$. (When $k_{j}=1$, we write $x_{I,j}\in F_{q}^{k_{j}\times I}$ as $x_{I,j}\in F_{q}^{1\times I}$ for notational simplicity.) Further, we let

$$x_{I}≜\left[\begin{array}{c} x_{I,1}\\ x_{I,2}\\ \vdots \\ x_{I,t} \end{array}\right]\in F_{q}^{k\times |I|},$$

called *the source matrix* generated by the source nodes in *I*. Clearly, we have $x_{I}=\left(x_{i}:\;\sigma_{i}\in I\right)$. Similarly, we use $F_{q}^{k\times I}$ (instead of $F_{q}^{k\times |I|}$ for notational simplicity) to denote the set of all possible k×|I| matrices taken by $x_{I}$. In particular, when I=S, we have

$$x_{S,j}=\left(x_{1,j},x_{2,j},\ldots ,x_{s,j}\right)\in F_{q}^{k_{j}\times S},\;\forall 1\leq j\leq t,$$

and

$$x_{S}=\left(x_{1},x_{2},\ldots ,x_{s}\right)\in F_{q}^{k\times S}.$$

Let $f_{1},f_{2},\ldots ,f_{t}$ be *t vector-linear functions* over a finite field $F_{q}$, called *target functions*. More precisely, for each 1≤j≤t, we let

$$f_{j}\left(x_{1,j},x_{2,j},\ldots ,x_{s,j}\right)=\left(x_{1,j},x_{2,j},\ldots ,x_{s,j}\right)\cdot M_{j},\;\forall x_{i,j}\in F_{q},1\leq i\leq s,$$

where $M_{j}$ is an $F_{q}$-valued column-full-rank matrix of size $s\times r_{j}$, i.e., $Rank\left(M_{j}\right)=r_{j}$ (which implies $r_{j}\leq s$). In our model, the sink node ρ demands to compute with zero error the target functions $f_{j}\left(x_{S,j}\right)$ for all 1≤j≤t, where $x_{S,j}$ is the *j*-th source submatrix generated by all the source nodes and

$$\begin{array}{c} f_{j}\left(x_{S,j}\right)≜x_{S,j}\cdot M_{j}=\left(x_{1,j},x_{2,j},\ldots ,x_{s,j}\right)\cdot M_{j}\in F_{q}^{k_{j}\times r_{j}},\;\forall 1\leq j\leq t. \end{array}$$

In other words, the sink node ρ is required to compute $f_{j}$, the target function, $k_{j}$ times with zero error for all 1≤j≤t. Then we have specified the network multi-function computation model, which is denoted by $\left(N,M_{j}:\;1\leq j\leq t\right)$.

In particular, when the sink node ρ is required to compute only a single target function with zero error (i.e., t=1), the above model degenerates to the network function computation model [1,2,3,4].

### 2.2. Network Multi-Function Computing Coding

In this subsection, we will define a *$\left(k_{j}:\;1\leq j\leq t;\;n\right)$ (network multi-function computing) code* for the model $\left(N,M_{j}:\;1\leq j\leq t\right)$. The purpose of such a code is to enable the sink node ρ to compute $f_{j}$, the target function, $k_{j}$ times with zero error for all j=1,2,…,t. To be specific, a $\left(k_{j}:\;1\leq j\leq t;\;n\right)$ code for $\left(N,M_{j}:\;1\leq j\leq t\right)$ consists of

- a *local encoding function* for each edge e∈E:
  (1) $$\begin{array}{c} \varphi_{e}:\left\{\begin{array}{cc} F_{q}^{k}\to F_{q}^{n} & \;if\;e\in Out(\sigma_{i})\;for\;some\;1\leq i\leq s,\\ \prod_{d\in In(tail(e))}F_{q}^{n}\to F_{q}^{n}\; & \;otherwise, \end{array}\right. \end{array}$$
  where $k=\sum_{j=1}^{t}k_{j};$
- *t decoding functions* $\psi_{j}$ with 1≤j≤t at the sink node ρ:
  $$\psi_{j}:\prod_{e\in In(\rho )}F_{q}^{n}\to F_{q}^{k_{j}\times r_{j}},\;\mathrm{for}\;\mathrm{all}\;j=1,2,\ldots ,t.$$

With the encoding mechanism as described, the local encoding functions $\varphi_{e},e\in E$ derive recursively the symbols transmitted over all edges *e*, denoted by $g_{e}\left(x_{S}\right)$, which can be considered as vectors in $F_{q}^{n}$. Specifically, $g_{e}$ can be written as
  (2) $$\begin{array}{c} g_{e}\left(x_{S}\right)=\left\{\begin{array}{cc} \varphi_{e}\left(x_{i}\right) & \;\mathrm{if}\;e\in \mathrm{Out}(\sigma_{i})\;for\;some\;1\leq i\leq s,\\ \varphi_{e}\left(g_{\mathrm{In}(u)}\left(x_{S}\right)\right) & \;otherwise, \end{array}\right. \end{array}$$
  where u=tail(e) and $g_{E}\left(x_{S}\right)≜\left(g_{e}\left(x_{S}\right):e\in E\right)$ for an edge set E⊆E. We call $g_{e}$ the *global encoding function* for an edge *e*.

We say that such a $\left(k_{j}:\;1\leq j\leq t;\;n\right)$ code $C=\left\{\varphi_{e}:e\in E;\psi_{j}:\;1\leq j\leq t\right\}$ is *admissible* if for all 1≤j≤t, the target function $f_{j}$ can be computed with zero error $k_{j}$ times at ρ by using C, i.e.,

$$\psi_{j}\left(g_{In(\rho )}\left(x_{S}\right)\right)=f_{j}\left(x_{S,j}\right)=x_{S,j}\cdot M_{j},\;\forall x_{S}\in F_{q}^{k\times S}.$$

To measure the performance of codes, we further define the *computing rate* for each target function $f_{j}$ with 1≤j≤t by

(3) $$\begin{array}{c} R_{j}\left(C\right)=\frac{k_{j}}{n}, \end{array}$$

describing the average number of times the target function $f_{j}$ can be computed with zero error at ρ for one use of the network N.

A *t*-tuple of nonnegative real numbers $\left(R_{j}:\;1\leq j\leq t\right)\in R_{+}^{t}$ is called *achievable* if ∀ϵ>0, there exists an admissible $\left(k_{j}:\;1\leq j\leq t;\;n\right)$ code C for the model $\left(N,M_{j}:\;1\leq j\leq t\right)$ such that

$$R_{j}<R_{j}\left(C\right)+\epsilon ,\;\forall 1\leq j\leq t.$$

Consequently, the rate region for the model $\left(N,M_{j}:\;1\leq j\leq t\right)$ is defined as

$$\begin{array}{c} R\left(N,M_{j}:\;1\leq j\leq t\right)≜\left\{\left(R_{1},R_{2},\ldots ,R_{t}\right)\in R_{+}^{t}:\left(R_{1},R_{2},\ldots ,R_{t}\right)is\;achievable\right\}, \end{array}$$

which is evidently closed and bounded.

## 3. Outer Bound on the Rate Region $R(N,\;M_{j}:\;1\leq j\leq t)$

In this section, we present a general outer bound on the rate region $R(N,\;M_{j}:\;1\leq j\leq t)$, where “general” means that the outer bound is applicable to arbitrary network topologies and arbitrary vector-linear functions. We first follow from [4] to present some graph-theoretic notations and definitions. Consider a network N=(G,S,ρ), where we recall that G=(V,E) is a directed acyclic graph. For two nodes u,v∈V, if there exists a directed path from *u* to *v* in N, we denote this relation by u→v. If there is no directed path from *u* to *v* in N, we say that *v* is *separated from u* and denote this relation by u↛v. Given a set of edges C⊆E, define $I_{C}$ as the set of the source nodes from which the sink node ρ is separated if *C* is deleted from E, i.e.,

$$I_{C}≜\left\{\sigma \in S:\rho \;\mathrm{is}\;\mathrm{separated}\;\mathrm{from}\;\sigma \;\mathrm{upon}\;\mathrm{deleting}\;\mathrm{the}\;\mathrm{edges}\;\mathrm{in}\;C\;\mathrm{from}\;E\right\}.$$

Equivalently, $I_{C}$ is the set of source nodes from which all directed paths to the sink node ρ pass through *C*. An edge set *C* is said to be a *cut set* if $I_{C}\neq \emptyset$. Further, we call *C* a *global cut* if $I_{C}=S$. We let Λ(N) denote the family of all cut sets in the network N, i.e.,

$$\Lambda \left(N\right)≜\left\{C\subseteq E:I_{C}\neq \emptyset \right\}.$$

Define a set $K_{C}$ for a cut set C∈Λ(N) as

$$K_{C}≜\left\{\sigma \in S:\exists e\in Cs.t.\sigma \to \mathrm{tail}(e)\right\}.$$

Then we can readily see that $K_{C}$ is the set of source nodes from which there exists a directed path to the sink node ρ that passes through *C*. It is clear that $I_{C}\subseteq K_{C}$. Further, we let $J_{C}=K_{C}\setminus I_{C}$, and hence $K_{C}=I_{C}\cup J_{C}$ and $I_{C}\cap J_{C}=\emptyset$. We note that if the set of edges *C* is given, $I_{C},J_{C}$ and $K_{C}$ are determined.

**Definition** **1** ([4], Definition 2, [14], Definition 3)**.** *Let C∈Λ(N) be a cut set and $P_{C}=\left\{C_{1},C_{2},\ldots ,C_{m}\right\}$ be a partition of the cut set C. The partition $P_{C}$ is said to be a strong partition of C if the following two conditions are satisfied:*

1. *$I_{C_{\ell }}\neq \emptyset$, ∀ℓ=1,2,…,m;*
2. *$I_{C_{i}}\cap K_{C_{j}}=\emptyset$, ∀1≤i,j≤m and i≠j. (There is a typo in the original definition of strong partition [4], Definition 2, where in 2), “$I_{C_{i}}\cap I_{C_{j}}=\emptyset$” in [4], Definition 2 should be “$I_{C_{i}}\cap K_{C_{j}}=\emptyset$” as stated in [14], Definition 3).*

For a cut set *C* in Λ(N), the partition {C} is called the *trivial strong partition* of *C*. Let C∈Λ(N) be an arbitrary cut set and $P_{C}=\left\{C_{1},C_{2},\ldots ,C_{m}\right\}$ be an arbitrary strong partition of *C*. For an $F_{q}$-valued matrix *M* of size s×r, we further define the *$P_{C}$-rank* of *M* by

(4) $${\mathrm{rank}}_{P_{C}}\left(M\right)≜Rank\left(M[I_{C}]\right)+\sum_{\ell =1}^{m}Rank\left(M[I_{C_{\ell }}]\right)-Rank\left(M[\cup_{\ell =1}^{m}I_{C_{\ell }}]\right),$$

where M[U] for a subset U⊆S stands for the submatrix of *M* containing the *i*th row if $\sigma_{i}\in U$. In particular, when $P_{C}=\left\{C\right\}$, the trivial strong partition of *C*, we have

$$\begin{array}{c} {\mathrm{rank}}_{\left\{C\right\}}\left(M\right)=Rank\left(M[I_{C}]\right). \end{array}$$

With the definition of the $P_{C}$-rank, we present in the following a general outer bound on the rate region, which can be applicable to arbitrary network topologies and arbitrary vector-linear functions. The proof of the outer bound is deferred to Section 3.

**Theorem** **1.** *Consider a model of network multi-function computation $\left(N,M_{j}:\;1\leq j\leq t\right)$. Then,*

$$\begin{array}{cc} \; & R\left(N,M_{j}:\;1\leq j\leq t\right)\\ & \subseteq \left\{\left(R_{1},R_{2},\ldots ,R_{t}\right)\in R_{+}^{t}:\sum_{j=1}^{t}{\mathrm{rank}}_{P_{C}}\left(M_{j}\right)\cdot R_{j}\leq \left|C\right|for\;all\left(C,P_{C}\right)\in \Lambda \left(N\right)\times P_{C}\right\}, \end{array}$$

*where $P_{C}$ denotes the collection of all the strong partitions of a cut set C∈Λ(N).*

The following example is given to illustrate the general outer bound obtained in Theorem 1.

**Figure 1:**
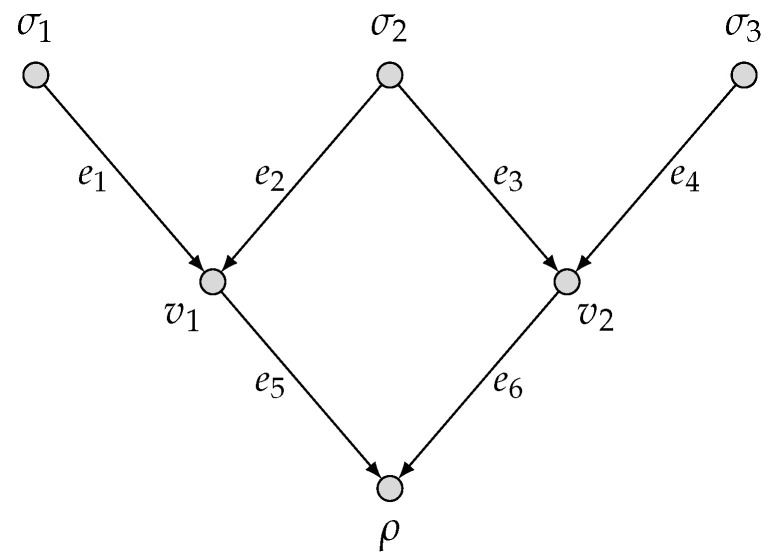
The network two-function computation model $\left(\tilde{N},\;M_{1},\;M_{2}\right)$.

**Example** **1.** *Consider a network two-function computation model $\left(\tilde{N},M_{1},M_{2}\right)$ as depicted in Figure 1, where in the diamond network $\tilde{N}$, there are three source nodes $\sigma_{1},\sigma_{2},\sigma_{3}$ and a single sink node ρ; and the two target functions $f_{1}$ and $f_{2}$ are specified below:*

$$\begin{array}{cc} f_{1}\left(x_{1,1},x_{2,1},x_{3,1}\right) & =x_{1,1}+x_{2,1}+x_{3,1},\;x_{1,1},x_{2,1},x_{3,1}\in F_{q},\\ f_{2}\left(x_{1,2},x_{2,2},x_{3,2}\right) & =\left(x_{1,2},x_{2,2},x_{3,2}\right),\;x_{1,2},x_{2,2},x_{3,2}\in F_{q}. \end{array}$$

*We readily see that the corresponding matrices of $f_{1}$ and $f_{2}$ are as follows:*

$$\begin{array}{c} M_{1}=\left[\begin{array}{c} 1\\ 1\\ 1 \end{array}\right]\;\mathrm{and}\;M_{2}=\left[\begin{array}{ccc} 1 & 0 & 0\\ 0 & 1 & 0\\ 0 & 0 & 1 \end{array}\right]. \end{array}$$

*We consider the global cut set $C=\{e_{5},e_{6}\}$, which has a unique nontrivial strong partition $P_{C}=\left\{C_{1}=\left\{e_{5}\right\},C_{2}=\left\{e_{6}\right\}\right\}$. We readily see that $I_{C}=S=\left\{\sigma_{1},\sigma_{2},\sigma_{3}\right\}$, $I_{C_{1}}=\left\{\sigma_{1}\right\}$, and $I_{C_{2}}=\left\{\sigma_{3}\right\}$. Then, we calculate that*

$$\begin{array}{cc} {\mathrm{rank}}_{P_{C}}\left(M_{1}\right) & =Rank\left(M_{1}\left[I_{C}\right]\right)+Rank\left(M_{1}\left[I_{C_{1}}\right]\right)+Rank\left(M_{1}\left[I_{C_{2}}\right]\right)-Rank\left(M_{1}\left[I_{C_{1}}\cup I_{C_{2}}\right]\right)\\ \; & =1+1+1-1=2. \end{array}$$

*Similarly, we calculate that*

$$\begin{array}{cc} {\mathrm{rank}}_{P_{C}}\left(M_{2}\right) & =Rank\left(M_{2}\left[I_{C}\right]\right)+Rank\left(M_{2}\left[I_{C_{1}}\right]\right)+Rank\left(M_{2}\left[I_{C_{2}}\right]\right)-Rank\left(M_{2}\left[I_{C_{1}}\cup I_{C_{2}}\right]\right)\\ \; & =3+1+1-2=3. \end{array}$$

*Then by Theorem 1, we obtain that*

(5) $$\begin{array}{cc} R(\tilde{N},M_{1},M_{2}) & \subseteq \left\{\left(R_{1},R_{2}\right)\in R_{+}^{2}:{\mathrm{rank}}_{P_{C}}\left(M_{1}\right)\cdot R_{1}+{\mathrm{rank}}_{P_{C}}\left(M_{2}\right)\cdot R_{2}\leq \left|C\right|\right\}\\ \; & =\left\{\left(R_{1},R_{2}\right)\in R_{+}^{2}:2R_{1}+3R_{2}\leq 2\right\}. \end{array}$$

*In fact, the outer bound in* (5) *is already tight, i.e.,*

$$\begin{array}{c} R\left(\tilde{N},M_{1},M_{2}\right)=\left\{\left(R_{1},R_{2}\right)\in R_{+}^{2}:2R_{1}+3R_{2}\leq 2\right\}, \end{array}$$

**Figure 2:**
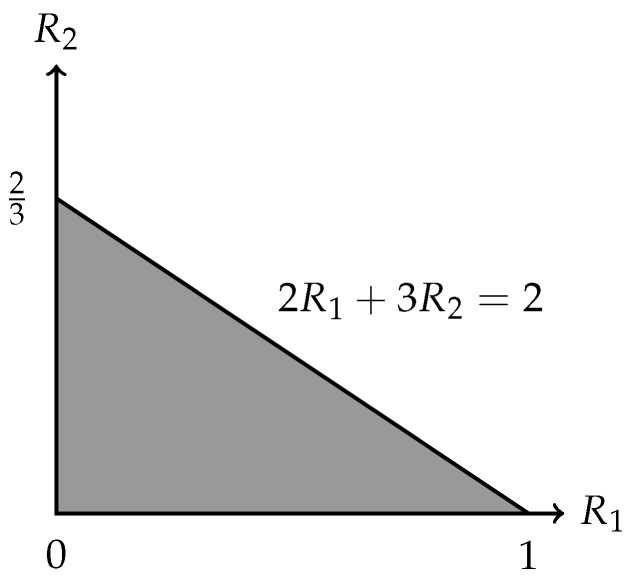
The rate region $R(\tilde{N},\;M_{1},\;M_{2})$.

*which is depicted in Figure 2. To establish this, it suffices to show that*

(6) $$\begin{array}{c} R\left(\tilde{N},M_{1},M_{2}\right)⊇\left\{\left(R_{1},R_{2}\right)\in R_{+}^{2}:2R_{1}+3R_{2}\leq 2\right\}. \end{array}$$

**Figure 3:**
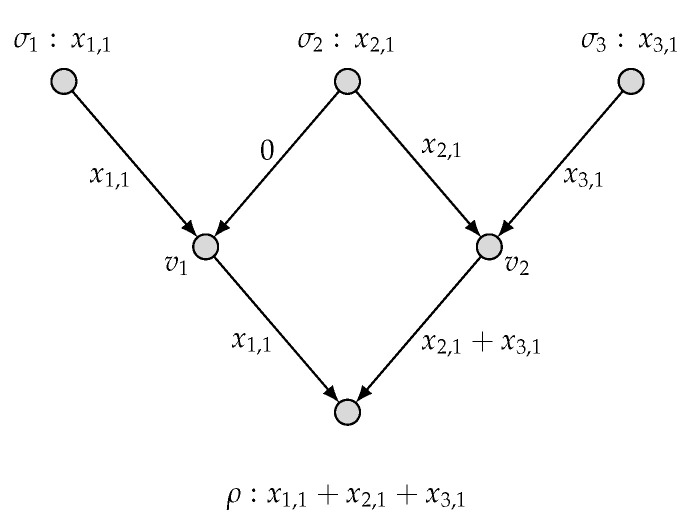
An admissible $\left(k_{1}=1,\;k_{2}=0;\;n=1\right)$ code for $\left(\tilde{N},\;M_{1},\;M_{2}\right)$.

**Figure 4:**
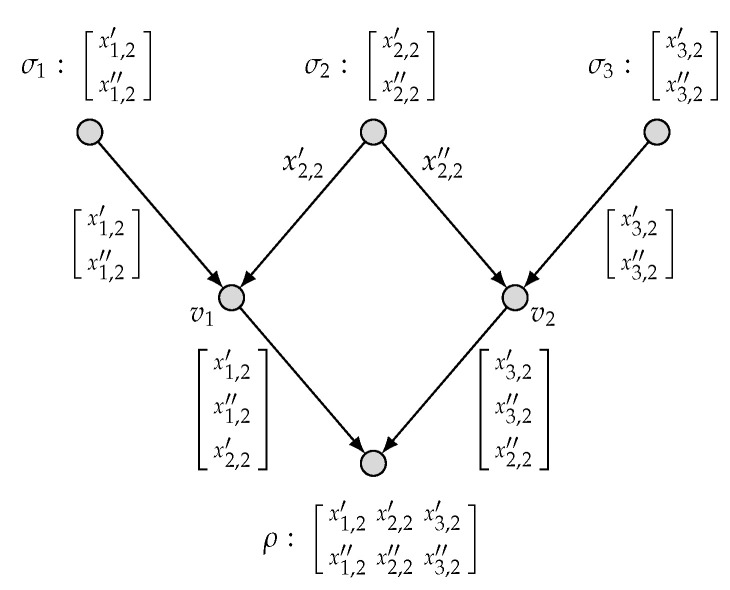
An admissible $\left(k_{1}=0,\;k_{2}=2;\;n=3\right)$ code for $\left(\tilde{N},\;M_{1},\;M_{2}\right)$.

*To be specific, we present in Figure 3 an admissible $\left(k_{1}=1,k_{2}=0;\;n=1\right)$ code $C^{\prime }$ and hence the computing rates $R_{1}\left(C^{\prime }\right)=1$ and $R_{2}\left(C^{\prime }\right)=0$. This implies that (1,0) is achievable. Similarly, we present in Figure 4 an admissible $\left(k_{1}=0,k_{2}=2;\;n=3\right)$ code $C^{\prime \prime }$ and hence the computing rates $R_{1}\left(C^{\prime \prime }\right)=0$ and $R_{2}\left(C^{\prime \prime }\right)=2/3$. This implies that (0,2/3) is achievable. By applying the time-sharing scheme, we thus have proved* (6).

### Proof of Theorem 1

In this subsection, we prove the outer bound in Theorem 1. First, we let $k_{1},k_{2},\ldots ,k_{t}$ be arbitrary *t* nonnegative integers, and accordingly define $k≜\sum_{p=1}^{t}k_{p}.$ (The index *p* is used here (instead of the original index *j*) to avoid potential symbol confusion, and this index notation will be consistently adopted in all subsequent proofs.) We then present the following equivalence relation, which will be used in the subsequent proof.

**Definition**  **2.** *Consider a subset of source nodes I⊆S. Let*

$$\begin{array}{c} x_{I}=\left[\begin{array}{c} x_{I,1}\\ x_{I,2}\\ \vdots \\ x_{I,t} \end{array}\right]\in F_{q}^{k\times I}\;\mathrm{and}\;x_{I}^{\prime }=\left[\begin{array}{c} x_{I,1}^{\prime }\\ x_{I,2}^{\prime }\\ \vdots \\ x_{I,t}^{\prime } \end{array}\right]\in F_{q}^{k\times I} \end{array}$$

*be any two source matrices, where $x_{I,p}$ and $x_{I,p}^{\prime }\in F_{q}^{k_{p}\times I}$ for 1≤p≤t. We say that $x_{I}$ and $x_{I}^{\prime }$ are I-equivalent if for each 1≤p≤t,*

$$x_{I,p}\cdot M_{p}\left[I\right]=x_{I,p}^{\prime }\cdot M_{p}\left[I\right].$$

The *I*-equivalence relation induces a partition of $F_{q}^{k\times I}$ and the blocks in the partition are called *I-equivalence classes*. We use ${\mathrm{Cl}}_{I}$ to denote an *I*-equivalence class. The following lemma establishes that all *I*-equivalence classes have the same size, and provides an explicit formula for this size.

**Lemma** **1.** *Consider a subset of source nodes I⊆S. All I-equivalence classes have the same size*

$$exp\left[\sum_{p=1}^{t}k_{p}\cdot \left(|I|-Rank(M_{p}\left[I\right])\right)\right],$$

*where exp[·] denotes the exponential function with base q, i.e., $exp\left[z\right]=q^{z}$.*

**Proof.**  See Appendix A. □

Immediately, we obtain the following consequence of Lemma 1, which explicitly gives the number of all *I*-equivalence classes.

**Corollary** **1.** *Consider a subset of source nodes I⊆S. The number of all I-equivalence classes is given by*

$$exp\left[\sum_{p=1}^{t}k_{p}\cdot Rank\left(M_{p}\left[I\right]\right)\right].$$

**Proof.**  We note that the *I*-equivalence relation induces a partition of $F_{q}^{k\times I}$, where $k=\sum_{p=1}^{t}k_{p}.$ In addition, by Lemma 1, all *I*-equivalence classes have the same size

$$exp\left[\sum_{p=1}^{t}k_{p}\cdot \left(|I|-Rank(M_{p}\left[I\right])\right)\right].$$

Then, the number of all *I*-equivalence classes is calculated to be

$$\begin{array}{cc} \; & \frac{\left|F_{q}^{k\times I}\right|}{exp\left[\sum_{p=1}^{t}k_{p}\cdot \left(|I|-Rank(M_{p}\left[I\right])\right)\right]}\\ & =\frac{exp\left[\sum_{p=1}^{t}k_{p}\cdot \left|I\right|\right]}{exp\left[\sum_{p=1}^{t}k_{p}\cdot \left(|I\right|-Rank\left(M_{p}\left[I\right]\right))\right]}=exp\left[\sum_{p=1}^{t}k_{p}\cdot Rank\left(M_{p}\left[I\right]\right)\right]. \end{array}$$

The corollary is proved. □

Now, we are ready to prove Theorem 1. Let C be an arbitrary admissible $\left(k_{p}:1\leq p\leq t;\;n\right)$ code for the model $\left(N,\;M_{p}:\;1\leq p\leq t\right)$, of which the global encoding functions are $g_{e},\;e\in E$. Consider a cut set C∈Λ(N), where we let $I=I_{C}$ and $J=J_{C}$. Since no directed path exists from any source node in S∖(I∪J) to any node in {tail(e):e∈C}, the source matrix $x_{S\setminus (I\cup J)}$ does not contribute to the value of $g_{C}\left(x_{S}\right)$. Hence, we can write $g_{C}\left(x_{I},x_{J},x_{S\setminus (I\cup J)}\right)$ as $g_{C}\left(x_{I},x_{J}\right)$. Next, we present the following lemma, which plays a crucial role in proving our general outer bound in Theorem 1.

**Lemma** **2.** *Let $\left\{g_{e}:e\in E\right\}$ be the set of all global encoding functions of a given admissible $\left(k_{p}:1\leq p\leq t;\;n\right)$ code for the model $\left(N,M_{p}:1\leq p\leq t\right)$ and $k=\sum_{p=1}^{t}k_{p}$. For a cut set $C\in \Lambda \left(N\right)$, let $I=I_{C}$ and $J=J_{C}$. Consider any two source matrices $x_{I}$ and $x_{I}^{\prime }$ in $F_{q}^{k\times I}$. If $x_{I}$ and $x_{I}^{\prime }$ are not I-equivalent, then*

$$g_{C}\left(x_{I},a_{J}\right)\neq g_{C}\left(x_{I}^{\prime },a_{J}\right),\;\forall a_{J}\in F_{q}^{k\times J}.$$

**Proof.**  See Appendix B. □

We recall the $\left(k_{p}:1\leq p\leq t;\;n\right)$ code C, of which the global encoding functions are $g_{e},\;e\in E$. We now consider a cut set C⊆Λ(N). Let $I=I_{C}$ and $J=J_{C}$ for notational simplicity. By (1) and (2), we can readily see that

(7) $$\begin{array}{cc} q^{n|C|} & \geq \#\left\{g_{C}\left(x_{I},x_{J}\right):\left(x_{I},x_{J}\right)\in F_{q}^{k\times (I\cup J)}\right\}\\ \; & \geq \#\left\{g_{C}\left(x_{I},a_{J}\right):x_{I}\in F_{q}^{k\times I}\right\}\\ \; & =\#\underset{\mathrm{all}\;{\mathrm{Cl}}_{I}}{⋃}\left\{g_{C}\left(x_{I},a_{J}\right):x_{I}\in {\mathrm{Cl}}_{I}\right\} \end{array}$$

(8) $$\begin{array}{cc} \; & =\sum_{\mathrm{all}\;{\mathrm{Cl}}_{I}}\#\left\{g_{C}\left(x_{I},a_{J}\right):x_{I}\in {\mathrm{Cl}}_{I}\right\}, \end{array}$$

where “#{·}” stands for the size of the set; $a_{J}\in F_{q}^{k\times J}$ is an arbitrarily fixed source matrix; the equality (7) follows from the fact that all *I*-equivalent classes ${\mathrm{Cl}}_{I}$ form a partition of $F_{q}^{k\times I}$; and the equality (8) follows from Lemma 2.

For each *I*-equivalent classes ${\mathrm{Cl}}_{I}$, we continue to consider

(9) $$\begin{array}{cc} \; & \#\left\{g_{C}\left(x_{I},a_{J}\right):x_{I}\in {\mathrm{Cl}}_{I}\right\}. \end{array}$$

For the cut set C∈Λ(N), let $P_{C}=\left\{C_{1},C_{2},\ldots ,C_{m}\right\}$ be an arbitrary strong partition of *C*. We further let $I_{\ell }=I_{C_{\ell }}$ for each 1≤ℓ≤m, and accordingly $L=I\setminus ({⋃}_{\ell =1}^{m}I_{\ell })$. By Definition 1, we can see that

$$\begin{array}{c} K_{C_{\ell }}\subseteq I_{\ell }\cup L\cup J,\;\forall 1\leq \ell \leq m. \end{array}$$

Then, we can rewrite (9) as follows:

(10) $$\begin{array}{c} \#\left\{g_{C}\left(x_{I},a_{J}\right):x_{I}\in {\mathrm{Cl}}_{I}\right\}\\ =\#\left\{\left(g_{C_{\ell }}\left(x_{I_{\ell }},x_{L},a_{J}\right):1\leq \ell \leq m\right):x_{I}=\left(x_{I_{1}},x_{I_{2}},\ldots ,x_{I_{m}},\;x_{L}\right)\in {\mathrm{Cl}}_{I}\right\}\\ \geq \#\{\left(g_{C_{\ell }}\left(x_{I_{\ell }},a_{L},a_{J}\right):1\leq \ell \leq m\right):x_{I_{\ell }}\in F_{q}^{k\times I_{\ell }},1\leq \ell \leq m,\\ \;\;\;\;\;\;\;\;\;\;\mathrm{and}\left(x_{I_{1}},x_{I_{2}},\ldots ,x_{I_{m}},\;a_{L}\right)\in {\mathrm{Cl}}_{I}\}, \end{array}$$

where we take $a_{L}\in F_{q}^{k\times L}$ as an arbitrary source matrix such that there exists a source matrix $\left(y_{I_{1}},y_{I_{2}},\ldots ,y_{I_{m}},y_{L}\right)\in {\mathrm{Cl}}_{I}$ with $y_{L}=a_{L}$.

We note that for the subset $I_{\ell }\subseteq I$ (where 1≤ℓ≤m), the $I_{\ell }$-equivalence relation similarly induces a partition of $F_{q}^{k\times I_{\ell }}$ and the blocks in the partition are called $I_{\ell }$-equivalence classes. For notational distinction, we use ${\mathrm{cl}}_{I_{\ell }}$ (instead of ${\mathrm{Cl}}_{I_{\ell }}$) to denote an $I_{\ell }$-equivalence class. For each $I_{\ell }$-equivalence class ${\mathrm{cl}}_{I_{\ell }}$, 1≤ℓ≤m, we define the set

$$\langle {\mathrm{cl}}_{I_{1}},{\mathrm{cl}}_{I_{2}},\ldots ,{\mathrm{cl}}_{I_{m}},a_{L}\rangle ≜\left\{\left(x_{I_{1}},x_{I_{2}},\ldots ,x_{I_{m}},a_{L}\right):x_{I_{\ell }}\in {\mathrm{cl}}_{I_{\ell }}\;\mathrm{for}\;1\leq \ell \leq m\right\}.$$

Continuing from (10), we consider

(11) $$\begin{array}{cc} \; & \;\;\;\;\#\{\left(g_{C_{\ell }}\left(x_{I_{\ell }},a_{L},a_{J}\right):1\leq \ell \leq m\right):x_{I_{\ell }}\in F_{q}^{k\times I_{\ell }},1\leq \ell \leq m,\\ \; & \;\;\;\;\;\;\;\mathrm{and}\left(x_{I_{1}},x_{I_{2}},\ldots ,x_{I_{m}},\;a_{L}\right)\in {\mathrm{Cl}}_{I}\}\\ =\; & \#\{\left(g_{C_{\ell }}\left(x_{I_{\ell }},a_{L},a_{J}\right):\;1\leq \ell \leq m\right):x_{I_{\ell }}\in {\mathrm{cl}}_{I_{\ell }},\mathrm{an}\;I_{\ell }-\mathrm{equivalence}\;\mathrm{class},1\leq \ell \leq m,\\ \; & \;\;\;\;\;\;\;\;\;\mathrm{and}\;\langle {\mathrm{cl}}_{I_{1}},{\mathrm{cl}}_{I_{2}},\ldots ,{\mathrm{cl}}_{I_{m}},a_{L}\rangle \subseteq {\mathrm{Cl}}_{I}\}\\ \geq \; & \#\{\langle {\mathrm{cl}}_{I_{1}},{\mathrm{cl}}_{I_{2}},\ldots ,{\mathrm{cl}}_{I_{m}},a_{L}\rangle :{\mathrm{cl}}_{I_{\ell }}\;\mathrm{is}\;\mathrm{an}\;I_{\ell }-\mathrm{equivalence}\;\mathrm{class},1\leq \ell \leq m,\\ \; & \;\;\;\;\;\;\;\;\;\;\mathrm{and}\;\langle {\mathrm{cl}}_{I_{1}},{\mathrm{cl}}_{I_{2}},\ldots ,{\mathrm{cl}}_{I_{m}},a_{L}\rangle \subseteq {\mathrm{Cl}}_{I}\}, \end{array}$$

where (11) is elaborated as follows. To be specific, let

$$\begin{array}{c} \mathrm{cl}≜\langle {\mathrm{cl}}_{I_{1}},{\mathrm{cl}}_{I_{2}},\ldots ,{\mathrm{cl}}_{I_{m}},a_{L}\rangle \subseteq {\mathrm{Cl}}_{I}\;\mathrm{and}\;{\mathrm{cl}}^{\prime }≜\langle {\mathrm{cl}}_{I_{1}}^{\prime },{\mathrm{cl}}_{I_{2}}^{\prime },\ldots ,{\mathrm{cl}}_{I_{m}}^{\prime },a_{L}\rangle \subseteq {\mathrm{Cl}}_{I} \end{array}$$

be any two distinct sets. We assume without loss of generality that ${\mathrm{cl}}_{I_{1}}\neq {\mathrm{cl}}_{I_{1}}^{\prime }$. Consider any two source matrices:

$$\begin{array}{c} \left(x_{I_{1}},x_{I_{2}},\ldots ,x_{I_{m}},\;a_{L}\right)\in \mathrm{cl}\;\mathrm{and}\;\left(x_{I_{1}}^{\prime },x_{I_{2}}^{\prime },\ldots ,x_{I_{m}}^{\prime },\;a_{L}\right)\in {\mathrm{cl}}^{\prime }, \end{array}$$

which implies that $x_{I_{\ell }}\in {\mathrm{cl}}_{I_{\ell }},x_{I_{\ell }}^{\prime }\in {\mathrm{cl}}_{I_{\ell }}^{\prime }$ for 1≤ℓ≤m. This, together with ${\mathrm{cl}}_{I_{1}}\neq {\mathrm{cl}}_{I_{1}}^{\prime }$, immediately implies that $x_{I_{1}}$ and $x_{I_{1}}^{\prime }$ are not $I_{1}$-equivalent. By the same way to prove Lemma 2, we have

$$g_{C_{1}}\left(x_{I_{1}},a_{L},a_{J}\right)\neq g_{C_{1}}\left(x_{I_{1}}^{\prime },a_{L},a_{J}\right),$$

and thus

$$\left(g_{C_{\ell }}\left(x_{I_{\ell }},a_{L},a_{J}\right):1\leq \ell \leq m\right)\neq \left(g_{C_{\ell }}\left(x_{I_{\ell }}^{\prime },a_{L},a_{J}\right):1\leq \ell \leq m\right).$$

Based on the above, the inequality (11) is proved.

Before discussing (11) further, we need the lemma below, whose proof is deferred to Appendix C.

**Lemma** **3.** *Consider a subset of source nodes I⊆S. Let $I_{\ell },1\leq \ell \leq m$ be m disjoint subsets of I and let $L=I\setminus (\cup_{\ell =1}^{m}I_{\ell })$. Fix an arbitrary I-equivalence class ${\mathrm{Cl}}_{I}$ and an arbitrary source matrix $a_{L}\in F_{q}^{k\times L}$ such that there exists a source matrix $y_{I}=\left(y_{I_{1}},y_{I_{2}},\ldots ,y_{I_{m}},y_{L}\right)\in {\mathrm{Cl}}_{I}$ with $y_{L}=a_{L}$. Let*

(12) $$\begin{array}{cc} P≜ & \{\langle {\mathrm{cl}}_{I_{1}},{\mathrm{cl}}_{I_{2}},\ldots ,{\mathrm{cl}}_{I_{m}},a_{L}\rangle :\;{\mathrm{cl}}_{I_{\ell }}\;\mathrm{is}\;\mathrm{an}\;I_{\ell }-\mathrm{equivalence}\;\mathrm{class},\;1\leq \ell \leq m,\\ \; & \;\;\;\;\;\;\mathrm{and}\langle {\mathrm{cl}}_{I_{1}},{\mathrm{cl}}_{I_{2}},\ldots ,{\mathrm{cl}}_{I_{m}},a_{L}\rangle \subseteq {\mathrm{Cl}}_{I}\}. \end{array}$$

*Then, the size of P is given by*

$$\begin{array}{c} \left|P\right|=exp\left[\sum_{p=1}^{t}k_{p}\cdot \left(\sum_{\ell =1}^{m}Rank\left(M_{p}\left[I_{\ell }\right]\right)-Rank\left(M_{p}\left[\cup_{\ell =1}^{m}I_{\ell }\right]\right)\right)\right]. \end{array}$$

With (10) and (11), by Lemma 3, we immediately obtain that

(13) $$\begin{array}{c} \#\left\{g_{C}\left(x_{I},a_{J}\right):x_{I}\in {\mathrm{Cl}}_{I}\right\}\geq exp\left[\sum_{p=1}^{t}k_{p}\cdot \left(\sum_{\ell =1}^{m}Rank\left(M_{p}\left[I_{\ell }\right]\right)-Rank\left(M_{p}\left[\cup_{\ell =1}^{m}I_{\ell }\right]\right)\right)\right]. \end{array}$$

Combining (8) and (13), we thus have

$$\begin{array}{cc} \; & q^{n|C|}\geq \sum_{\mathrm{all}\;{\mathrm{Cl}}_{I}}exp\left[\sum_{p=1}^{t}k_{p}\cdot \left(\sum_{\ell =1}^{m}Rank\left(M_{p}\left[I_{\ell }\right]\right)-Rank\left(M_{p}\left[\cup_{\ell =1}^{m}I_{\ell }\right]\right)\right)\right] \end{array}$$

(14) $$\begin{array}{cc} \; & =exp\left[\sum_{p=1}^{t}k_{p}\cdot Rank\left(M_{p}\left[I\right]\right)\right]\cdot exp\left[\sum_{p=1}^{t}k_{p}\cdot \left(\sum_{\ell =1}^{m}Rank\left(M_{p}\left[I_{\ell }\right]\right)-Rank\left(M_{p}\left[\cup_{\ell =1}^{m}I_{\ell }\right]\right)\right)\right]\\ \; & =exp\left[\sum_{p=1}^{t}k_{p}\cdot \left(Rank\left(M_{p}\left[I\right]\right)+\sum_{\ell =1}^{m}Rank\left(M_{p}\left[I_{\ell }\right]\right)-Rank\left(M_{p}\left[\cup_{\ell =1}^{m}I_{\ell }\right]\right)\right)\right] \end{array}$$

(15) $$\begin{array}{cc} \; & =exp\left[\sum_{p=1}^{t}k_{p}\cdot {\mathrm{rank}}_{P_{C}}\left(M_{p}\right)\right], \end{array}$$

where the equality (14) follows from Corollary 1, and the equality (15) follows from the definition of ${\mathrm{rank}}_{P_{C}}\left(M_{p}\right)$ for 1≤p≤t (cf. (4)). Equivalently, we have

(16) $$\begin{array}{c} n|C|\geq \sum_{p=1}^{t}k_{p}\cdot {\mathrm{rank}}_{P_{C}}\left(M_{p}\right). \end{array}$$

It further follows from (16) that

(17) $$\begin{array}{cc} \left|C\right| & \geq \sum_{p=1}^{t}\;\frac{k_{p}}{n}\cdot {\mathrm{rank}}_{P_{C}}\left(M_{p}\right)=\sum_{p=1}^{t}R_{p}\left(C\right)\cdot {\mathrm{rank}}_{P_{C}}\left(M_{p}\right), \end{array}$$

where we recall in (3) that the computing rate for the target function $f_{p}$ is defined by $R_{p}\left(C\right)=k_{p}/n$ for each 1≤p≤t. We note that the inequality (17) is true for all pairs $\left(C,P_{C}\right)\in \Lambda \left(N\right)\times P_{C}$, and thus

$$\begin{array}{c} \sum_{p=1}^{t}{\mathrm{rank}}_{P_{C}}\left(M_{p}\right)\cdot R_{p}\left(C\right)\leq \left|C\right|,\;\forall \left(C,P_{C}\right)\in \Lambda \left(N\right)\times P_{C}. \end{array}$$

Further, the above outer bound is valid for any *t* nonnegative integers $k_{1},k_{2},\ldots ,k_{t}$, and any admissible $\left(k_{p}:1\leq p\leq t;\;n\right)$ code. Hence, we have proved that

$$\begin{array}{cc} \; & R(N,M_{p}:\;1\leq p\leq t)\\ & \subseteq \left\{\left(R_{1},R_{2},\ldots ,R_{t}\right)\in R_{+}^{t}:\sum_{p=1}^{t}{\mathrm{rank}}_{P_{C}}\left(M_{p}\right)\cdot R_{p}\leq \left|C\right|for\;all\left(C,P_{C}\right)\in \Lambda \left(N\right)\times P_{C}\right\}. \end{array}$$

The theorem is proved.

## 4. Comparison on Network Function Computation

The model of network function computation (N,f) for computing an arbitrary target function *f* over a directed acyclic network N has been investigated persistently in the literature [1,2,3,4,5,6,7,8,9,10,11,12,13,14,15,16]. In the same way, we can define the rate region R(N,f) for the model (N,f). Further, the computing capacity of the model (N,f) is defined as

$$\begin{array}{c} C(N,f)≜\max\;R(N,f), \end{array}$$

namely that the maximum average number of times that the target function *f* can be computed with zero error for one use of the network. We first present the following theorem, which reveals the relationship between the network multi-function computation rate region and the network function computation rate region.

**Theorem** **2.** *Consider the model of network multi-function computation $\left(N,M_{j}:\;1\leq j\leq t\right)$ and the models of network function computation $\left(N,M_{j}\right)$ for 1≤j≤t. On the one hand,*

(18) $$\begin{array}{c} R\left(N,M_{j}:\;1\leq j\leq t\right)\subseteq \left\{\left(R_{1},R_{2},\ldots ,R_{t}\right):R_{j}\in R\left(N,M_{j}\right)for1\leq j\leq t\right\}. \end{array}$$

*On the other hand, the following inclusion holds:*

(19) $$\begin{array}{cc} \; & R\left(N,M_{j}:\;1\leq j\leq t\right)⊇\{\sum_{j=1}^{t}\lambda_{j}\cdot R_{j}e_{j}=\left(\lambda_{1}R_{1},\lambda_{2}R_{2},\ldots ,\lambda_{t}R_{t}\right):\\ \; & \;\;\;\;\;\;\;\;R_{j}\in R\left(N,M_{j}\right)and\lambda_{j}\geq 0for1\leq j\leq t,and\sum_{j=1}^{t}\lambda_{j}\leq 1\}, \end{array}$$

*where $e_{j}$ is a t-dimensional row vector whose j-th component is* 1*, and all other components are* 0*.*

**Proof.**  See Appendix D. □

**Remark** **1.** *We note that the inclusions* (18) *and* (19) *in Theorem 2 are in general not tight. To prove this claim, we will use a specific model presented in Example 2.*

In network function computation, several “general” upper bounds on the computing capacity have been obtained [1,2,4], where “general” means that the upper bounds are applicable to arbitrary networks and arbitrary target functions. Here, the best known upper bound is the one proved by Guang et al. [4] in using the approach of the cut-set strong partition. Notably, this best-known upper bound also equivalently provides the best outer bound on the rate region. In particular, the best-known upper bound on the computing capacity in ([4], Theorem 2) for an arbitrary network and an arbitrary vector-linear function can be verified as follows, and its equivalent form explicitly characterizes the best outer bound on the rate region.

**Theorem** **3.** *Consider a model of network function computation (N,M). Then,*

$$\begin{array}{c} C\left(N,M\right)\leq \min_{\left(C,P_{C}\right)\in \Lambda \left(N\right)\times P_{C}}\;\frac{\left|C\right|}{{\mathrm{rank}}_{P_{C}}\left(M\right)}, \end{array}$$

*or equivalently,*

$$\begin{array}{cc} \; & R\left(N,M\right)\subseteq \left\{R\in R_{+}:R\leq \min_{\left(C,P_{C}\right)\in \Lambda \left(N\right)\times P_{C}}\;\frac{\left|C\right|}{{\mathrm{rank}}_{P_{C}}\left(M\right)}\right\}. \end{array}$$

We note that Theorem 3 is a straightforward consequence of Theorem 1. On the other hand, we show in the following that Theorem 3, which gives the outer bound on the network function computation rate region, can induce an outer bound on the network multi-function computation rate region. However, this outer bound is not as tight as the one given by Theorem 1.

By Theorem 3 and (18) in Theorem 2, we consider the model of network multi-function computation, and then obtain that

$$\begin{array}{cc} \; & R\left(N,M_{j}:\;1\leq j\leq t\right)\subseteq \left\{\left(R_{1},R_{2},\ldots ,R_{t}\right):R_{j}\in R\left(N,M_{j}\right)for1\leq j\leq t\right\}\\ \; & \subseteq \left\{\left(R_{1},R_{2},\ldots ,R_{t}\right)\in R_{+}^{t}:R_{j}\leq \min_{\left(C,P_{C}\right)\in \Lambda \left(N\right)\times P_{C}}\;\frac{\left|C\right|}{{\mathrm{rank}}_{P_{C}}\left(M_{j}\right)}for1\leq j\leq t\right\}. \end{array}$$

We formally state this outer bound on the rate region for the model of network multi-function computation $\left(N,M_{j}:\;1\leq j\leq t\right)$ in the following theorem.

**Theorem** **4.** *Consider a model of network multi-function computation $\left(N,\;M_{j}:\;1\leq j\leq t\right)$. Then,*

$$\begin{array}{cc} \; & R(N,\;M_{j}:\;1\leq j\leq t)\\ & \subseteq \left\{\left(R_{1},R_{2},\ldots ,R_{t}\right)\in R_{+}^{t}:R_{j}\leq \min_{\left(C,P_{C}\right)\in \Lambda \left(N\right)\times P_{C}}\;\frac{\left|C\right|}{{\mathrm{rank}}_{P_{C}}\left(M_{j}\right)}for1\leq j\leq t\right\}. \end{array}$$

We first note that our outer bound in Theorem 1 is tighter than the outer bound in Theorem 4 induced by Theorem 3. To be specific, we have

$$\begin{array}{cc} \; & \left\{\left(R_{1},R_{2},\ldots ,R_{t}\right)\in R_{+}^{t}:\sum_{j=1}^{t}{\mathrm{rank}}_{P_{C}}\left(M_{j}\right)\cdot R_{j}\leq \left|C\right|for\;all\left(C,P_{C}\right)\in \Lambda \left(N\right)\times P_{C}\right\}\\ \; & \subseteq \{\left(R_{1},R_{2},\ldots ,R_{t}\right)\in R_{+}^{t}:{\mathrm{rank}}_{P_{C}}\left(M_{j}\right)\cdot R_{j}\leq \left|C\right|.\\ \; & \;\;\;\;\;\;\;.forall\left(C,P_{C}\right)\in \Lambda \left(N\right)\times P_{C}and1\leq j\leq t\}\\ \; & =\left\{\left(R_{1},R_{2},\ldots ,R_{t}\right)\in R_{+}^{t}:R_{j}\leq \min_{\left(C,P_{C}\right)\in \Lambda \left(N\right)\times P_{C}}\;\frac{\left|C\right|}{{\mathrm{rank}}_{P_{C}}\left(M_{j}\right)}for1\leq j\leq t\right\}. \end{array}$$

Further, we use the specific example below to illustrate that our outer bound is a strict enhancement of the outer bound in Theorem 4.

**Figure 5:**
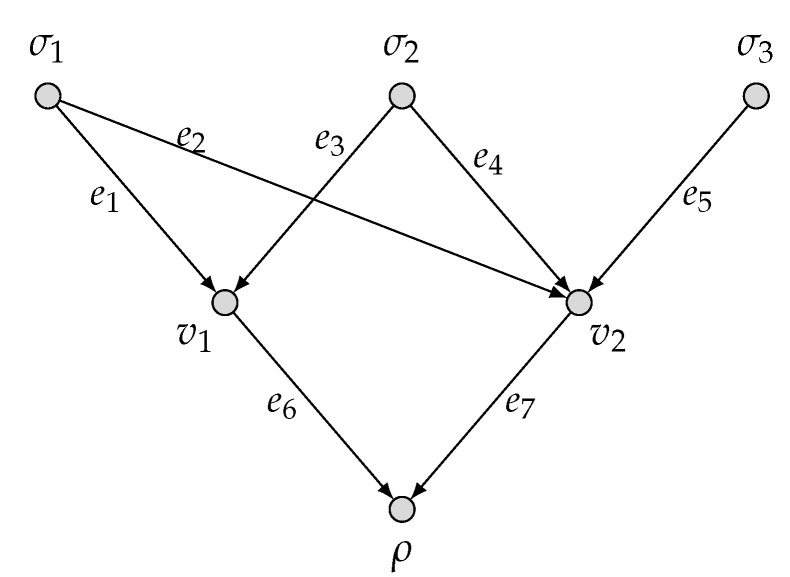
The network two-function computation model $\left(\hat{N},\;M_{1},\;M_{2}\right)$.

**Example** **2.** *Consider a network two-function computation model $\left(\hat{N},M_{1},M_{2}\right)$ as depicted in Figure 5, where in the asymmetric diamond network $\hat{N}$, there are three source nodes $\sigma_{1},\sigma_{2},\sigma_{3}$ and a single sink node ρ; and the two target functions $f_{1}$ and $f_{2}$ are specified below:*

$$\begin{array}{cc} f_{1}\left(x_{1,1},x_{2,1},x_{3,1}\right) & =x_{1,1}+x_{2,1}+x_{3,1},\;x_{1,1},x_{2,1},x_{3,1}\in F_{q},\\ f_{2}\left(x_{1,2},x_{2,2},x_{3,2}\right) & =\left(x_{1,2},x_{2,2},x_{3,2}\right),\;x_{1,2},x_{2,2},x_{3,2}\in F_{q}. \end{array}$$

*We readily see that the corresponding matrices of $f_{1}$ and $f_{2}$ are as follows:*

$$\begin{array}{c} M_{1}=\left[\begin{array}{c} 1\\ 1\\ 1 \end{array}\right]\;\mathrm{and}\;M_{2}=\left[\begin{array}{ccc} 1 & 0 & 0\\ 0 & 1 & 0\\ 0 & 0 & 1 \end{array}\right]. \end{array}$$

*We will calculate the outer bound in Theorem 1 by considering two typical cut sets and their strong partitions below.*

- *We first consider the cut set $C=\{e_{5}\}$ and its trivial strong partition $P_{C}=\left\{C\right\}=\left\{\{e_{5}\}\right\}$. We can see that $I_{C}=\left\{\sigma_{3}\right\}$, and thus*
  (20) $$\begin{array}{c} {\mathrm{rank}}_{P_{C}}\left(M_{j}\right)=Rank\left(M_{j}\left[I_{C}\right]\right)=1,\;j=1,2. \end{array}$$
  *By Theorem 1, we have*
  (21) $$\begin{array}{cc} R(\hat{N},M_{1},M_{2}) & \subseteq \left\{\left(R_{1},R_{2}\right)\in R_{+}^{2}:{\mathrm{rank}}_{P_{C}}\left(M_{1}\right)\cdot R_{1}+{\mathrm{rank}}_{P_{C}}\left(M_{2}\right)\cdot R_{2}\leq \left|C\right|\right\}\\ \; & =\left\{\left(R_{1},R_{2}\right)\in R_{+}^{2}:R_{1}+R_{2}\leq 1\right\}. \end{array}$$
- *In the following, we consider the global cut set $C=\{e_{6},e_{7}\}$ and its trivial strong partition $P_{C}=\left\{C\right\}=\left\{\{e_{6},e_{7}\}\right\}$. We can see that $I_{C}=S$, and thus*
  (22) $$\begin{array}{c} {\mathrm{rank}}_{P_{C}}\left(M_{1}\right)=Rank\left(M_{1}\left[I_{C}\right]\right)=1\;and\;{\mathrm{rank}}_{P_{C}}\left(M_{2}\right)=Rank\left(M_{2}\left[I_{C}\right]\right)=3. \end{array}$$
  *By Theorem 1, we also have*
  (23) $$\begin{array}{cc} R(\hat{N},M_{1},M_{2}) & \subseteq \left\{\left(R_{1},R_{2}\right)\in R_{+}^{2}:{\mathrm{rank}}_{P_{C}}\left(M_{1}\right)\cdot R_{1}+{\mathrm{rank}}_{P_{C}}\left(M_{2}\right)\cdot R_{2}\leq \left|C\right|\right\}\\ \; & =\left\{\left(R_{1},R_{2}\right)\in R_{+}^{2}:R_{1}+3R_{2}\leq 2\right\}. \end{array}$$

*Combining* (21) *and* (23)*, we thus obtain that*

(24) $$\begin{array}{c} R\left(\hat{N},M_{1},M_{2}\right)\subseteq \left\{\left(R_{1},R_{2}\right)\in R_{+}^{2}:R_{1}+R_{2}\leq 1,2R_{1}+3R_{2}\leq 2\right\}. \end{array}$$

*In fact, the outer bound in* (24) *is already tight, i.e.,*

(25) $$\begin{array}{c} R\left(\hat{N},M_{1},M_{2}\right)=\left\{\left(R_{1},R_{2}\right)\in R_{+}^{2}:R_{1}+R_{2}\leq 1,2R_{1}+3R_{2}\leq 2\right\}, \end{array}$$

**Figure 6:**
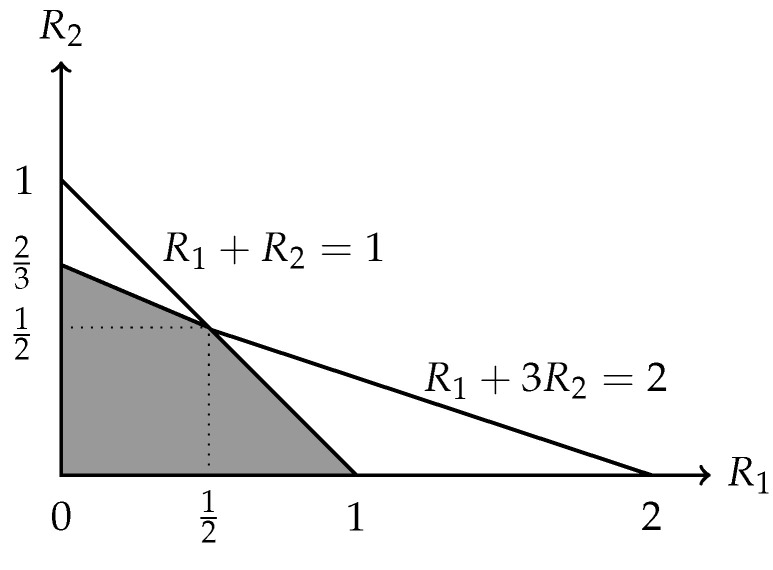
The rate region $R(\hat{N},\;M_{1},\;M_{2})$.

*which is depicted in Figure 6. To establish this, it suffices to show that*

(26) $$\begin{array}{c} R\left(\hat{N},M_{1},M_{2}\right)⊇\left\{\left(R_{1},R_{2}\right)\in R_{+}^{2}:R_{1}+R_{2}\leq 1,2R_{1}+3R_{2}\leq 2\right\}. \end{array}$$

**Figure 7:**
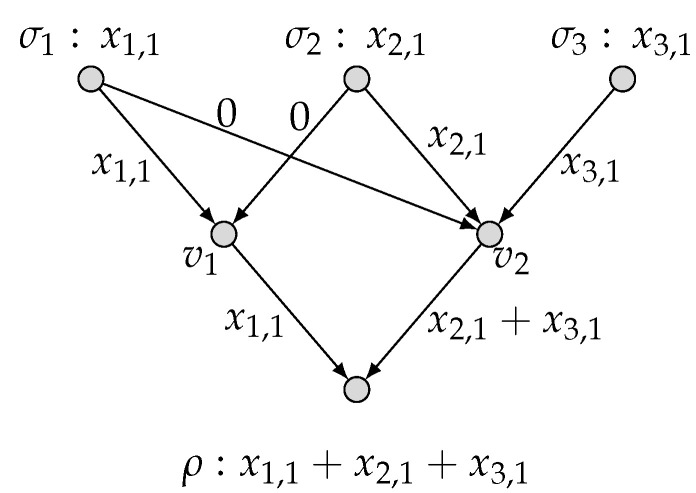
An admissible $\left(k_{1}=1,\;k_{2}=0;\;n=1\right)$ code for $\left(\hat{N},\;M_{1},\;M_{2}\right)$.

**Figure 8:**
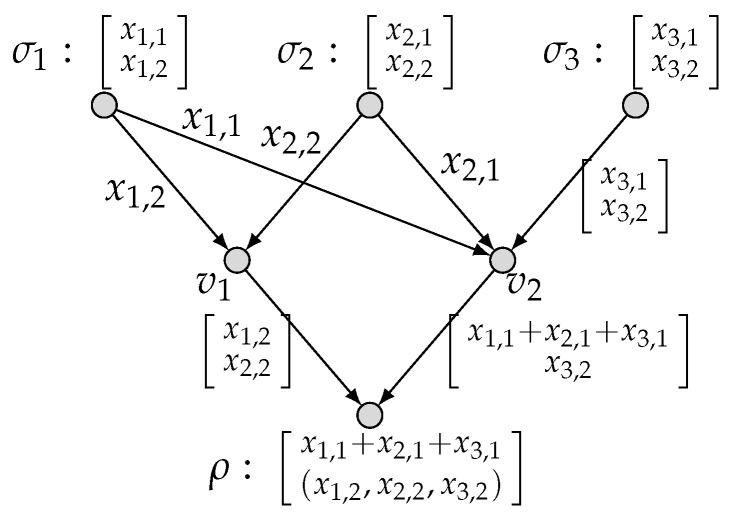
An admissible $\left(k_{1}=1,\;k_{2}=1;\;n=2\right)$ code for $\left(\hat{N},\;M_{1},\;M_{2}\right)$.

**Figure 9:**
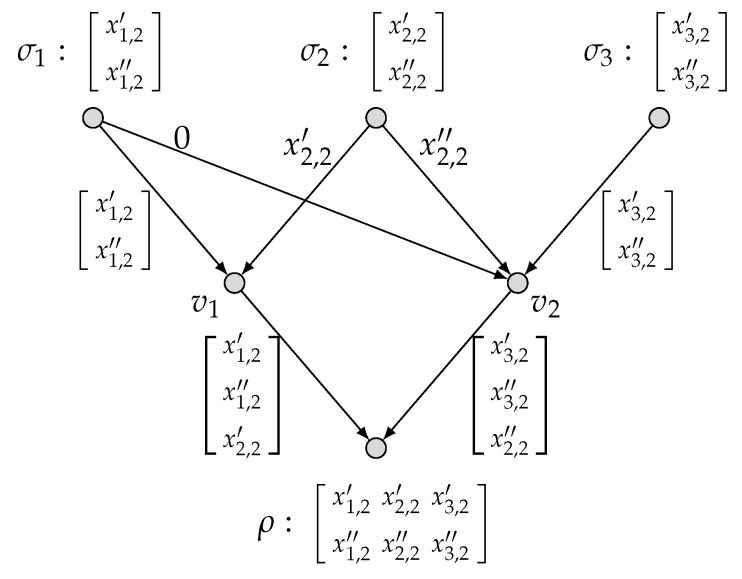
An admissible $\left(k_{1}=0,\;k_{2}=2;\;n=3\right)$ code for $\left(\hat{N},\;M_{1},\;M_{2}\right)$.

*To be specific, we present in Figure 7 an admissible $\left(k_{1}=1,k_{2}=0;\;n=1\right)$ code $C^{\prime }$ and hence the computing rates $R_{1}\left(C^{\prime }\right)=1$ and $R_{2}\left(C^{\prime }\right)=0$. This implies that (1,0) is achievable. Further, we present in Figure 8 an admissible $\left(k_{1}=1,k_{2}=1;\;n=2\right)$ code $C^{\prime \prime }$ and hence the computing rates $R_{1}\left(C^{\prime \prime }\right)=1/2$ and $R_{2}\left(C^{\prime \prime }\right)=1/2$. This implies that (1/2,1/2) is achievable. Similarly, we present in Figure 9 an admissible $\left(k_{1}=0,k_{2}=2;\;n=3\right)$ code $C^{\prime \prime \prime }$ and hence the computing rates $R_{1}\left(C^{\prime \prime \prime }\right)=0$ and $R_{2}\left(C^{\prime \prime \prime }\right)=2/3$. This implies that (0,2/3) is achievable. By applying the time-sharing scheme, we show that* (26) *holds. Together with* (24) *, we thus have proved* (25)*.*

*In the following, we calculate the outer bound in Theorem 4. First, we have*

$$\begin{array}{c} 1\leq C\left(\hat{N},M_{1}\right)\leq \min_{\left(C,P_{C}\right)\in \Lambda \left(\hat{N}\right)\times P_{C}}\;\frac{\left|C\right|}{{\mathrm{rank}}_{P_{C}}\left(M_{1}\right)}\leq \frac{\left|\{e_{5}\}\right|}{{\mathrm{rank}}_{\left\{\left\{e_{5}\right\}\right\}}\left(M_{1}\right)}=1, \end{array}$$

*where the first inequality holds because there exists an admissible (1;1) code for the model $\left(\hat{N},M_{1}\right)$ as depicted in Figure 7; the second inequality follows from Theorem 3; and the last equality follows from ${\mathrm{rank}}_{\left\{\left\{e_{5}\right\}\right\}}\left(M_{1}\right)=1$ by* (20)*. This implies that*

(27) $$\begin{array}{c} C\left(\hat{N},M_{1}\right)=\min_{\left(C,P_{C}\right)\in \Lambda \left(\hat{N}\right)\times P_{C}}\;\frac{\left|C\right|}{{\mathrm{rank}}_{P_{C}}\left(M_{1}\right)}=1. \end{array}$$

*Similarly, we also have*

$$\begin{array}{c} \frac{2}{3}\leq C\left(\hat{N},\;M_{2}\right)\leq \min_{\left(C,P_{C}\right)\in \Lambda \left(\hat{N}\right)\times P_{C}}\;\frac{\left|C\right|}{{\mathrm{rank}}_{P_{C}}\left(M_{2}\right)}\leq \frac{\left|\{e_{6},e_{7}\}\right|}{{\mathrm{rank}}_{\left\{\left\{e_{6},e_{7}\right\}\right\}}\left(M_{2}\right)}=\frac{2}{3}, \end{array}$$

*where the first inequality holds because there exists an admissible (2;3) code for the model $\left(\hat{N},M_{2}\right)$ as depicted in Figure 9; and the last equality follows from ${\mathrm{rank}}_{\left\{\left\{e_{6},e_{7}\right\}\right\}}\left(M_{2}\right)=3$ by* (22)*. This implies that*

(28) $$\begin{array}{c} C\left(\hat{N},M_{2}\right)=\min_{\left(C,P_{C}\right)\in \Lambda \left(\hat{N}\right)\times P_{C}}\;\frac{\left|C\right|}{{\mathrm{rank}}_{P_{C}}\left(M_{2}\right)}=\frac{2}{3}. \end{array}$$

*By* (27) *and* (28)*, we obtain the outer bound in Theorem 4 as follows:*

(29) $$\begin{array}{c} R\left(\hat{N},M_{1},M_{2}\right)\subseteq \left\{\left(R_{1},R_{2}\right)\in R_{+}^{2}:R_{1}\leq 1,R_{2}\leq \frac{2}{3}\right\}. \end{array}$$

*We can readily see that the outer bound in* (25) *is strictly tighter than the outer bound in* (29)*. This thus shows that our outer bound in Theorem 1 is a strict enhancement of the outer bound in Theorem 4.*

*In the end of the example, we prove the claim in Remark 1, i.e., the inclusions* (18) *and* (19) *in Theorem 2 are in general not tight. First, it follows from* (27) *and* (28) *that*

$$\begin{array}{cc} \; & \left\{\left(R_{1},R_{2}\right):R_{1}\in R\left(\hat{N},M_{1}\right)\;\mathrm{and}\;R_{2}\in R\left(\hat{N},M_{2}\right)\right\}\\ & =\left\{\left(R_{1},R_{2}\right)\in R_{+}^{2}:R_{1}\leq 1,R_{2}\leq \frac{2}{3}\right\}⊃R\left(\hat{N},M_{1},M_{2}\right), \end{array}$$

*where the last inequality follows from* (25)*. This thus shows that the inclusion* (18) *in Theorem 2 is in general not tight.*

*By* (27) *and* (28)*, we also have*

$$\begin{array}{cc} \; & \left\{\lambda_{1}\cdot \left(R_{1},0\right)+\lambda_{2}\cdot \left(0,R_{2}\right):R_{j}\in R\left(\hat{N},M_{j}\right)\;\mathrm{and}\;\lambda_{j}\geq 0\;\mathrm{for}\;j=1,2,\;\mathrm{and}\;\lambda_{1}+\lambda_{2}\leq 1\right\}\\ \; & =\left\{\left(\lambda_{1}\cdot R_{1},\lambda_{2}\cdot R_{2}\right):0\leq R_{1}\leq 1,0\leq R_{2}\leq \frac{2}{3},\lambda_{1}\geq 0,\lambda_{2}\geq 0,\;\mathrm{and}\;\lambda_{1}+\lambda_{2}\leq 1\right\}\\ & =\left\{\left({\bar{R}}_{1},{\bar{R}}_{2}\right)\in R_{+}^{2}:{\bar{R}}_{1}+\frac{3}{2}{\bar{R}}_{2}\leq 1\right\}\subset R\left(\hat{N},M_{1},M_{2}\right), \end{array}$$

*which implies that the inclusion* (19) *in Theorem 2 is in general not tight.*

We note that Example 2 plays a key role. On the one hand, this example demonstrates that the outer bound in Theorem 1 is strictly tighter than the induced outer bound in Theorem 4. On the other hand, this example illustrates that the network multi-function computation rate region cannot be directly derived from network function computation rate regions by the time-sharing scheme. This further underscores that studying the network multi-function computation offers advantages over separately investigating the network function computations.

## 5. Conclusions

In this paper, we put forward the problem of multi-function computation over a directed acyclic network. We proved an outer bound on the rate region by developing the approach of the cut-set strong partition introduced by Guang et al. We also illustrated that the obtained outer bound is tight for a typical model of computing two vector-linear functions over the diamond network. Furthermore, we compared network multi-function computation and network function computation. We first established the relationship between the network multi-function rate region and the network function computation rate region. By this relationship, we showed that the best known outer bound on the network function computation rate region can induce an outer bound on the network multi-function computation rate region. However, this induced outer bound is not as tight as our outer bound. Further, we showed that the best known outer bound on the rate region for computing an arbitrary vector-linear function over an arbitrary network is a straightforward consequence of our outer bound.

For the network multi-function computation model considered in the current paper, several interesting problems still remain open, such as whether the presented outer bound on the rate region is tight, and whether the used method can be generalized to characterize the outer bound on the rate region for more general functions.

## Data Availability

The original contributions presented in this study are included in the article.

## Appendix A. Proof of Lemma 1

For a subset of source nodes I⊆S, we let ${\mathrm{Cl}}_{I}\subseteq F_{q}^{k\times I}$ be an arbitrary *I*-equivalence class, where we recall that $k=\sum_{p=1}^{t}k_{p}.$ For any source matrix $x_{I}\in F_{q}^{k\times I}$, we write

$$\begin{array}{c} x_{I}=\left[\begin{array}{c} x_{I,1}\\ x_{I,2}\\ \vdots \\ x_{I,t} \end{array}\right]\in F_{q}^{k\times I} \end{array}$$

with $x_{I,p}\in F_{q}^{k_{p}\times I}$ for 1≤p≤t. Further, we take an arbitrarily fixed source matrix in ${\mathrm{Cl}}_{I}$:

$$\begin{array}{c} a_{I}=\left[\begin{array}{c} a_{I,1}\\ a_{I,2}\\ \vdots \\ a_{I,t} \end{array}\right]\in {\mathrm{Cl}}_{I}. \end{array}$$

Then, by Definition 2, the *I*-equivalence class ${\mathrm{Cl}}_{I}$ can be written as:

$$\begin{array}{cc} {\mathrm{Cl}}_{I} & =\left\{x_{I}\in F_{q}^{k\times I}:x_{I,p}\cdot M_{p}\left[I\right]=a_{I,p}\cdot M_{p}\left[I\right]\;\mathrm{for}\;\mathrm{all}\;1\leq p\leq t\right\}. \end{array}$$

We thus obtain that

$$\begin{array}{cc} |{\mathrm{Cl}}_{I}| & =\#\left\{x_{I}\in F_{q}^{k\times I}:x_{I,p}\cdot M_{p}\left[I\right]=a_{I,p}\cdot M_{p}\left[I\right]\;\mathrm{for}\;\mathrm{all}\;1\leq p\leq t\right\}\\ \; & =\prod_{p=1}^{t}\#\left\{x_{I,p}\in F_{q}^{k_{p}\times I}:x_{I,p}\cdot M_{p}\left[I\right]=a_{I,p}\cdot M_{p}\left[I\right]\right\}\\ \; & =\prod_{p=1}^{t}exp\left[k_{p}\cdot \left(|I|-Rank(M_{p}\left[I\right])\right)\right]\\ & =exp\left[\sum_{p=1}^{t}k_{p}\cdot \left(|I|-Rank(M_{p}\left[I\right])\right)\right]. \end{array}$$

The lemma is proved.

## Appendix B. Proof of Lemma 2

Let $\left\{g_{e}:e\in E\right\}$ be the set of all global encoding functions of a given admissible $\left(k_{p}:1\leq p\leq t;\;n\right)$ code C for the model $\left(N,M_{p}:1\leq p\leq t\right)$. We further let C∈Λ(N) be a cut set. To simplify the notation, we let $I=I_{C}$ and $J=J_{C}$. Consider any two source matrices that are not *I*-equivalent:

$$\begin{array}{c} x_{I}=\left[\begin{array}{c} x_{I,1}\\ x_{I,2}\\ \vdots \\ x_{I,t} \end{array}\right]\in F_{q}^{k\times I}\;\mathrm{and}\;x_{I}^{\prime }=\left[\begin{array}{c} x_{I,1}^{\prime }\\ x_{I,2}^{\prime }\\ \vdots \\ x_{I,t}^{\prime } \end{array}\right]\in F_{q}^{k\times I}, \end{array}$$

where $x_{I,p},x_{I,p}^{\prime }\in F_{q}^{k_{p}\times I}$ for 1≤p≤t are the row blocks of $x_{I}$ and $x_{I}^{\prime }$, respectively. By Definition 2, we assume without loss of generality that

(A1) $$x_{I,1}\cdot M_{1}\left[I\right]\neq x_{I,1}^{\prime }\cdot M_{1}\left[I\right].$$

In the following, we take

$$\begin{array}{c} a_{J}=\left[\begin{array}{c} a_{J,1}\\ a_{J,2}\\ \vdots \\ a_{J,t} \end{array}\right]\in F_{q}^{k\times J}\;\mathrm{and}\;d=\left[\begin{array}{c} d_{1}\\ d_{2}\\ \vdots \\ d_{t} \end{array}\right]\in F_{q}^{k\times S\setminus (I\cup J)}, \end{array}$$

such that all $a_{J,p}\in F_{q}^{k_{p}\times J}$ and $d_{p}\in F_{q}^{k_{p}\times S\setminus \left(I\cup J\right)}$ for 1≤p≤k are arbitrary. Then, the inequality (A1) implies that

(A2) $$\begin{array}{cc} \; & x_{I,1}\cdot M_{1}\left[I\right]+a_{J,1}\cdot M_{1}\left[J\right]+d_{1}\cdot M_{1}\left[S\setminus \left(I\cup J\right)\right]\\ & \neq x_{I,1}^{\prime }\cdot M_{1}\left[I\right]+a_{J,1}\cdot M_{1}\left[J\right]+d_{1}\cdot M_{1}\left[S\setminus \left(I\cup J\right)\right]. \end{array}$$

Let $D={⋃}_{\sigma \in (S\setminus I)}\mathrm{Out}\left(\sigma \right)$, an edge subset of E. Then $\hat{C}≜C\cup D$ is a global cut set, i.e., $I_{\hat{C}}=S$. Since $g_{In(\rho )}$ is a function of $g_{\hat{C}}$ and the code C can compute all target functions with zero error, by (A2), we obtain that

(A3) $$g_{\hat{C}}\left(x_{I},\;a_{J},d\right)\neq g_{\hat{C}}\left(x_{I}^{\prime },\;a_{J},\;d\right).$$

We further note that $\hat{C}=C\cup D$, $K_{C}=I\cup J$ and $K_{D}=S\setminus I$. Then, we can rewrite (A3) as follows:

$$\begin{array}{cc} \left(g_{C}\left(x_{I},a_{J}\right),\;g_{D}\left(a_{J},d\right)\right) & =g_{\hat{C}}\left(x_{I},a_{J},d\right)\neq g_{\hat{C}}\left(x_{I}^{\prime },a_{J},d\right)=\left(g_{C}\left(x_{I}^{\prime },a_{J}\right),\;g_{D}\left(a_{J},d\right)\right). \end{array}$$

This immediately implies that $g_{C}\left(x_{I},a_{J}\right)\neq g_{C}\left(x_{I}^{\prime },a_{J}\right).$ The lemma is proved.

## Appendix C. Proof of Lemma 3

Consider a subset of source nodes I⊆S. Let $I_{\ell },1\leq \ell \leq m$ be *m* disjoint subsets of *I* and let $L=I\setminus (\cup_{\ell =1}^{m}I_{\ell })$. We let ${\mathrm{Cl}}_{I}\subseteq F_{q}^{k\times I}$ be an arbitrary *I*-equivalence class and $a_{L}\in F_{q}^{k\times L}$ be an arbitrary source matrix such that there exists a source matrix $y_{I}=\left(y_{I_{1}},y_{I_{2}},\ldots ,y_{I_{m}},y_{L}\right)\in {\mathrm{Cl}}_{I}$ with $y_{L}=a_{L}$, where we recall that $k=\sum_{p=1}^{t}k_{p}.$ We further let ${\mathrm{Cl}}_{I}\left(a_{L}\right)$ be the set formed by all source matrices $x_{I}=\left(x_{I_{1}},x_{I_{2}},\ldots ,x_{I_{m}},x_{L}\right)\in {\mathrm{Cl}}_{I}$ with $x_{L}=a_{L}$, namely that

(A4) $$\begin{array}{c} {\mathrm{Cl}}_{I}\left(a_{L}\right)≜\left\{x_{I}=\left(x_{I_{1}},x_{I_{2}},\ldots ,x_{I_{m}},x_{L}\right)\in {\mathrm{Cl}}_{I}:x_{L}=a_{L}\right\}. \end{array}$$

Before discussing further, we need the lemma below, whose proof is deferred to the end of the appendix.

**Lemma** **A1.** *Consider an I-equivalence class ${\mathrm{Cl}}_{I}$. Then, P (cf.* (12)*) is a partition of ${\mathrm{Cl}}_{I}\left(a_{L}\right)$, namely that all the sets $\langle {\mathrm{cl}}_{I_{1}},{\mathrm{cl}}_{I_{2}},\ldots ,{\mathrm{cl}}_{I_{m}},a_{L}\rangle$ with ${\mathrm{cl}}_{I_{\ell }}$ being an $I_{\ell }$-equivalence class for 1≤ℓ≤m that satisfy $\langle {\mathrm{cl}}_{I_{1}},{\mathrm{cl}}_{I_{2}},\ldots ,{\mathrm{cl}}_{I_{m}},a_{L}\rangle \subseteq {\mathrm{Cl}}_{I}$ constitute a partition of ${\mathrm{Cl}}_{I}\left(a_{L}\right)$.*

In the following, we focus on the size of the set ${\mathrm{Cl}}_{I}\left(a_{L}\right)$. For any source matrices $x_{I}\in F_{q}^{k\times I}$, $x_{I_{\ell }}\in F_{q}^{k\times I_{\ell }}$ (where 1≤ℓ≤m), and $x_{L}\in F_{q}^{k\times L}$, we write

$$\begin{array}{c} x_{I}=\left[\begin{array}{c} x_{I,1}\\ x_{I,2}\\ \vdots \\ x_{I,t} \end{array}\right]\in F_{q}^{k\times I},\;x_{I_{\ell }}=\left[\begin{array}{c} x_{I_{\ell },1}\\ x_{I_{\ell },2}\\ \vdots \\ x_{I_{\ell },t} \end{array}\right]\in F_{q}^{k\times I_{\ell }},\;\mathrm{and}\;x_{L}=\left[\begin{array}{c} x_{L,1}\\ x_{L,2}\\ \vdots \\ x_{L,t} \end{array}\right]\in F_{q}^{k\times L} \end{array}$$

with $x_{I,p}\in F_{q}^{k_{p}\times I},x_{I_{\ell },p}\in F_{q}^{k_{p}\times I_{\ell }},x_{L,p}\in F_{q}^{k_{p}\times L}$ for 1≤p≤t. Further, we take an arbitrarily fixed source matrix in ${\mathrm{Cl}}_{I}$:

$$\begin{array}{c} a_{I}=\left[\begin{array}{c} a_{I,1}\\ a_{I,2}\\ \vdots \\ a_{I,t} \end{array}\right]\in {\mathrm{Cl}}_{I}. \end{array}$$

Then by Definition 2, the *I*-equivalence class ${\mathrm{Cl}}_{I}$ can be written as:

(A5) $$\begin{array}{c} {\mathrm{Cl}}_{I}=\left\{x_{I}\in F_{q}^{k\times I}:x_{I,p}\cdot M_{p}\left[I\right]=a_{I,p}\cdot M_{p}\left[I\right]\;\mathrm{for}\;\mathrm{all}\;1\leq p\leq t\right\}. \end{array}$$

Combining (A4) and (A5), we further have

(A6) $$\begin{array}{cc} \; & {\mathrm{Cl}}_{I}\left(a_{L}\right)=\{x_{I}=\left(x_{I_{1}},x_{I_{2}},\ldots ,x_{I_{m}},x_{L}\right)\in F_{q}^{k\times I}:x_{L}=a_{L}\;\mathrm{and}\;x_{I,p}\cdot M_{p}\left[I\right]=a_{I,p}\cdot M_{p}\left[I\right]\\ \; & \;\;\;\;\;\;\;\;\;\;\;\;\;\;\;\;\;\;\mathrm{for}\;\mathrm{all}\;1\leq p\leq t\}\\ \; & =\{x_{I}=\left(x_{I_{1}},x_{I_{2}},\ldots ,x_{I_{m}},x_{L}\right)\in F_{q}^{k\times I}:x_{L}=a_{L}\;\mathrm{and}\;\\ \; & \;\left(x_{I_{1},p},x_{I_{2},p},\ldots ,x_{I_{m},p}\right)\cdot M_{p}\left[\cup_{\ell =1}^{m}I_{\ell }\right]+x_{L,p}\cdot M_{p}\left[L\right]=a_{I,p}\cdot M_{p}\left[I\right]\;\mathrm{for}\;\mathrm{all}\;1\leq p\leq t\}\\ \; & =\{\left(x_{I_{1}},x_{I_{2}},\ldots ,x_{I_{m}}\right)\in F_{q}^{k\times (\cup_{\ell =1}^{m}I_{\ell })}:\\ \; & \;\left(x_{I_{1},p},x_{I_{2},p},\ldots ,x_{I_{m},p}\right)\cdot M_{p}\left[\cup_{\ell =1}^{m}I_{\ell }\right]+a_{L,p}\cdot M_{p}\left[L\right]=a_{I,p}\cdot M_{p}\left[I\right]\;\mathrm{for}\;\mathrm{all}\;1\leq p\leq t\}. \end{array}$$

By (A6), we can compute the size of the set ${\mathrm{Cl}}_{I}\left(a_{L}\right)$ as follows:

(A7) $$\begin{array}{cc} \left|{\mathrm{Cl}}_{I}\left(a_{L}\right)\right| & =\prod_{p=1}^{t}\#\{\left(x_{I_{1},p},x_{I_{2},p},\ldots ,x_{I_{m},p}\right)\in F_{q}^{k_{p}\times \left(\cup_{\ell =1}^{m}I_{\ell }\right)}:\\ \; & \;\;\;\;\;\;\left(x_{I_{1},p},x_{I_{2},p},\ldots ,x_{I_{m},p}\right)\cdot M_{p}\left[\cup_{\ell =1}^{m}I_{\ell }\right]+a_{L,p}\cdot M_{p}\left[L\right]=a_{I,p}\cdot M_{p}\left[I\right]\}\\ \; & =\prod_{p=1}^{t}exp\left[k_{p}\cdot \left(\sum_{\ell =1}^{m}\left|I_{\ell }\right|-Rank\left(M_{p}\left[\cup_{\ell =1}^{m}I_{\ell }\right]\right)\right)\right]\\ \; & =exp\left[\sum_{p=1}^{t}k_{p}\cdot \left(\sum_{\ell =1}^{m}\left|I_{\ell }\right|-Rank\left(M_{p}\left[\cup_{\ell =1}^{m}I_{\ell }\right]\right)\right)\right]. \end{array}$$

In addition, by Lemma A1, P is a partition of ${\mathrm{Cl}}_{I}\left(a_{L}\right)$. We also note that by Lemma 1, all $I_{\ell }$-equivalence (1≤ℓ≤m) classes have the same size

$$exp\left[\sum_{p=1}^{t}k_{p}\cdot \left(|I_{\ell }|-Rank\left(M_{p}\left[I_{\ell }\right]\right)\right)\right].$$

Then, for any set $\mathrm{cl}=\langle {\mathrm{cl}}_{I_{1}},{\mathrm{cl}}_{I_{2}},\ldots ,{\mathrm{cl}}_{I_{m}},a_{L}\rangle \in P$ with ${\mathrm{cl}}_{I_{\ell }}$ being an $I_{\ell }$-equivalence class for 1≤ℓ≤m, its size is given by

(A8) $$\begin{array}{c} \left|\mathrm{cl}\right|=\prod_{\ell =1}^{m}exp\left[\sum_{p=1}^{t}k_{p}\cdot \left(|I_{\ell }|-Rank\left(M_{p}\left[I_{\ell }\right]\right)\right)\right]=exp\left[\sum_{p=1}^{t}k_{p}\cdot \sum_{\ell =1}^{m}\left(|I_{\ell }|-Rank\left(M_{p}\left[I_{\ell }\right]\right)\right)\right]. \end{array}$$

By (A7) and (A8), the size of P is calculated to be

$$\begin{array}{cc} \left|P\right| & =\frac{exp\left[\sum_{p=1}^{t}k_{p}\cdot \left(\sum_{\ell =1}^{m}\left|I_{\ell }\right|-Rank\left(M_{p}\left[\cup_{\ell =1}^{m}I_{\ell }\right]\right)\right)\right]}{exp\left[\sum_{p=1}^{t}k_{p}\cdot \sum_{\ell =1}^{m}\left(|I_{\ell }|-Rank\left(M_{p}\left[I_{\ell }\right]\right)\right)\right]}\\ \; & =exp\left[\sum_{p=1}^{t}k_{p}\cdot \left(\sum_{\ell =1}^{m}Rank\left(M_{p}\left[I_{\ell }\right]\right)-Rank\left(M_{p}\left[\cup_{\ell =1}^{m}I_{\ell }\right]\right)\right)\right]. \end{array}$$

To complete the proof of Lemma 3, it remains to prove Lemma A1.

**Proof** **of** **Lemma** **A1.** Consider the *I*-equivalence class ${\mathrm{Cl}}_{I}$ and an arbitrary source matrix $a_{L}\in F_{q}^{k\times L}$ such that there exists a source matrix $y_{I}=\left(y_{I_{1}},y_{I_{2}},\ldots ,y_{I_{m}},y_{L}\right)\in {\mathrm{Cl}}_{I}$ with $y_{L}=a_{L}$. First, we can readily see that all the sets in P (cf. (12)) are disjoint and ${⋃}_{\mathrm{cl}\in P}\mathrm{cl}\subseteq {\mathrm{Cl}}_{I}\left(a_{L}\right).$ To complete the proof, it suffices to prove that ${⋃}_{\mathrm{cl}\in P}\mathrm{cl}⊇{\mathrm{Cl}}_{I}\left(a_{L}\right).$ We consider an arbitrary source matrix $x_{I}=\left(x_{I_{1}},\;x_{I_{2}},\;\ldots ,\;x_{I_{m}},\;a_{L}\right)$ in ${\mathrm{Cl}}_{I}\left(a_{L}\right)$. Clearly, $x_{I}\in {\mathrm{Cl}}_{I}$. Next, we will prove $x_{I}\in \mathrm{cl}$ for some cl∈P. First, we recall that for each 1≤ℓ≤m, $F_{q}^{k\times I_{\ell }}$ can be partitioned into $I_{\ell }$-equivalence classes. Hence, $x_{I_{\ell }}$ is in some $I_{\ell }$-equivalence class, say ${\mathrm{cl}}_{I_{\ell }}$, and then

(A9) $$\begin{array}{c} x_{I}=\left(x_{I_{1}},\;x_{I_{2}},\;\ldots ,\;x_{I_{m}},\;a_{L}\right)\in \mathrm{cl}≜\langle {\mathrm{cl}}_{I_{1}},{\mathrm{cl}}_{I_{2}},\ldots ,{\mathrm{cl}}_{I_{m}},a_{L}\rangle . \end{array}$$

We can verify that all source matrices in cl are *I*-equivalent. As such, there always exists an *I*-equivalence class ${\mathrm{Cl}}_{I}^{\prime }$ such that

(A10) $$\begin{array}{c} \mathrm{cl}=\langle {\mathrm{cl}}_{I_{1}},{\mathrm{cl}}_{I_{2}},\ldots ,{\mathrm{cl}}_{I_{m}},a_{L}\rangle \subseteq {\mathrm{Cl}}_{I}^{\prime }, \end{array}$$

and further $\mathrm{cl}\subseteq {\mathrm{Cl}}_{I}^{\prime }\left(a_{L}\right)$. Combining (A9) and (A10), we have $x_{I}\in {\mathrm{Cl}}_{I}^{\prime }\left(a_{L}\right)\subseteq {\mathrm{Cl}}^{\prime }.$ Together with $x_{I}\in {\mathrm{Cl}}_{I}\left(a_{L}\right)\subseteq \mathrm{Cl}$, we immediately obtain that $\mathrm{Cl}={\mathrm{Cl}}^{\prime }$ and also ${\mathrm{Cl}}_{I}^{\prime }\left(a_{L}\right)={\mathrm{Cl}}_{I}\left(a_{L}\right)$. This thus implies that $\mathrm{cl}\subseteq {\mathrm{Cl}}_{I}\left(a_{L}\right)$, i.e., cl∈P, and hence $x_{I}\in {⋃}_{\mathrm{cl}\in P}\mathrm{cl}.$ As such, we have proved ${\mathrm{Cl}}_{I}\left(a_{L}\right)\subseteq {⋃}_{\mathrm{cl}\in P}\mathrm{cl}$ and the lemma is proved. □

## Appendix D. Proof of Theorem 2

We first note that an admissible code for the model $\left(N,M_{j}:\;1\leq j\leq t\right)$ can also be regarded as an admissible code for the network function computation model $\left(N,M_{j}\right)$, where 1≤j≤t. This immediately shows that

$$\begin{array}{c} R\left(N,M_{j}:\;1\leq j\leq t\right)\subseteq \left\{\left(R_{1},R_{2},\ldots ,R_{t}\right):R_{j}\in R\left(N,M_{j}\right)for1\leq j\leq t\right\}. \end{array}$$

In the following, we prove the inclusion (19). For each index 1≤j≤t, we let $R_{j}\in R\left(N,M_{j}\right)$, namely that the target function $f_{j}$ can be computed with zero error $R_{j}$ times on average for one use of the network N. This immediately implies that $R_{j}e_{j}\in R\left(N,M_{j}:\;1\leq j\leq t\right).$ We further let $\lambda_{j}\geq 0$ for 1≤j≤t, and $\sum_{j=1}^{t}\lambda_{j}\leq 1$. By the time-sharing scheme, we have

(A11) $$\sum_{j=1}^{t}\lambda_{j}\cdot R_{j}e_{j}\in R\left(N,M_{j}:\;1\leq j\leq t\right),$$

namely that all target functions $f_{j}$ (1≤j≤t) can be computed with zero error $\lambda_{j}R_{j}$ times for one use of the network N, respectively. We note that Equation (A11) holds for any $R_{j}\in R\left(N,M_{j}\right)$ and $\lambda_{j}\geq 0$ for 1≤j≤t, and $\sum_{j=1}^{t}\lambda_{j}\leq 1$, This immediately implies that

$$\begin{array}{cc} \; & R(N,M_{j}:\;1\leq j\leq t)\\ & ⊇\left\{\sum_{j=1}^{t}\lambda_{j}\cdot R_{j}e_{j}:R_{j}\in R\left(N,M_{j}\right)and\lambda_{j}\geq 0for1\leq j\leq t,and\sum_{j=1}^{t}\lambda_{j}\leq 1\right\}. \end{array}$$

The theorem is proved.
